# Aphids Facing Their Parasitoids: A First Look at How Chemical Signals May Make Higher Densities of the Pea Aphid *Acyrthosiphon pisum* Less Attractive to the Parasitoid *Aphidius ervi*

**DOI:** 10.3390/insects12100878

**Published:** 2021-09-28

**Authors:** Mohannad Ismail, Penelope Zanolli, Frédéric Muratori, Thierry Hance

**Affiliations:** 1 Biodiversity Research Centre, Earth and Life Institute, Université Catholique de Louvain, Croix du Sud 4-5, 1348 Louvain-la-Neuve, Belgium; penelope.zanolli@libero.it (P.Z.); frederic.muratori@gmail.com (F.M.); thierry.hance@uclouvain.be (T.H.); 2 Dipartimento di Scienze Agrarie e Ambientali, Università di Udine, Via Delle Scienze 208, 33100 Udine, Italy

**Keywords:** behavior, bet hedging strategy, bottom-up, density, 6-methyl-5-hepten-2-one, vigilance

## Abstract

**Simple Summary:**

Successful foraging behavior of parasitoids depends on specific organic information emitted by host-infested plants. For instance, the emission of volatile compounds increases in infested plants, and these are the first indicator of host presence. Parasitoids are attracted by these volatiles in a quite specific way. By combining behavioral and chemical studies, we showed bottom-up effects in a broad bean *Vicia faba* (Fabaceae)–pea aphid *Acyrthosiphon pisum* (Homoptera: Aphididae)–parasitoid *Aphidius ervi* (Hymenoptera: Braconidae) model system. We found that behavioral selection of parasitoid females toward plants with a high density of aphid infestation was reduced, and this can be linked to reduced emission of volatile compounds. In practice, if parasitoids are less attracted to plants with high-density aphid infestations, there may be potential negative impacts on biological control. Therefore, the common recommendation in biological control is to release parasitoids early in the season when aphid density on crop plants is still low.

**Abstract:**

Herbivore-induced plant volatiles constitute the first indicators of insect host presence, and these can affect the foraging behavior of their natural enemies. The density of insect hosts may affect the nature and concentration of these plant-induced volatiles. We tested the impact of infestation density (low, intermediate, and high) of the pea aphid, *Acyrthosiphon pisum* (Homoptera: Aphididae), feeding on the broad bean *Vicia faba*, on the attractiveness of the parasitoid *Aphidius ervi* (Hymenoptera: Braconidae), using a Y-tube olfactometer (infested vs. non-infested plants). The emitted volatile compounds from both infested and non-infested plants were collected and identified. In addition, two series of experiments were carried out to test the impact of the presence of a conspecific female parasitoid within the aphid/plant complex on the attractiveness to other females. Parasitoids were significantly more attracted to the plants with low and intermediate aphid infestation levels. The volatile blend composition of the infested plants changed in relation to aphid density and may explain the low attraction of parasitoids toward high aphid density. The presence of conspecific females on the aphid patch had no apparent impact on the behavioral choices of other parasitoid females. Our study adds a new aspect to understanding plant–aphid–parasitoid interactions, including the possibility that aphids may manipulate chemical cues of host plants affecting the orientation of parasitoids.

## 1. Introduction

The predator–prey or parasitoid–host relationships in ecology consist of the interactions between two species and their subsequent effects on each other. In such interactions, members of the higher trophic level feed on members of the lower level, affecting the population growth of each. While the higher trophic levels search for better predation/parasitism opportunities, the lower levels adapt to be more defensive [1]. The most well-known hypothesis in animal defense behavior is the group vigilance hypothesis [2], also referred to as the “many eyes effect” [3], well studied in many bird [4], mammal [5], and fish species [6]. The hypothesis contends that group living can reduce the risk of being encountered by predators [7], increase the time of feeding by lowering the frequency of scanning the environment, dilute the risk of being attacked [8], and decrease the attack efficiency by confusing the predator [9]. However, living in groups could attract predators more, especially among prey species with less capacity for defense [10].

Similarly, insects have adapted and developed various strategies to decrease predation risk [11,12]. Aphids are small sap-sucking, gregarious insects that feed on a wide range of plants [13] and are attacked by many natural enemies, including parasitoids. Parasitoid species are used worldwide in biological control programs against agricultural pests [14,15,16]. Aphids have developed many strategies to escape the attack of parasitoids, including direct resistance behaviors (kicking, swinging the body), escape behaviors (dropping from plants), or indirect defense [17,18,19]. Indirect defense in aphids involves chemical responses, such as releasing alarm pheromones to alert other individuals and colonies [20,21]. However, aphid colony size has largely not been considered and is poorly studied as a defense strategy.

Group size defense has been studied on various insects. For instance, Foster and Treherne [8] found that the attack rate of the fish *Sardinops sagas* Jenyns (Clupeidae) on the marine insect *Halobates robustus* Barber (Hemiptera: Gerridae) decreased with increasing group size of *H. robustus*. In addition, Treherne and Foster [22] demonstrated that individuals of *H. robustus* quickly responded when they were in large groups by escaping when the fish approached.

In order to achieve successful parasitism, parasitoids need to locate and recognize their hosts [23]. During the host foraging process, parasitoids have to distinguish a broad spectrum of cues in the form of complex odors emitted from undamaged plants, infested plants, and host herbivore insects [24,25]. Plants exhibit indirect plant defenses against insect herbivores, whereby plants attract parasitoids and predators by releasing specific compounds, such as the green leaf volatiles or the herbivore-induced plant volatiles (HIPVs) into the surrounding air. In most cases, HIPVs are the first indicators that help parasitoid foragers to locate their hosts over long distances [26,27,28,29]. Once parasitoids have landed on infested plants, they start to use other visual, mechanical, and chemical cues in the close-contact micro-habitat, such as size, shape, color, and density of herbivore insects [30,31,32].

While the composition of the HIPVs shows almost no change with the density of herbivore insects on the plant, the amounts of volatiles may increase with herbivore density [33,34,35]. For instance, Shiojiri et al. [36] found that the seedlings of a cabbage (*Brassica oleracea* var. capitata, cv Shikidori) released greater amounts of volatiles when attacked by an increasing number of larvae of the cabbage white butterfly *Pieris rapae* Linnaeus (Lepidoptera: Pieridae), and thus attracted the parasitoid *Cotesia glomerata* Linnaeus (Hymenoptera: Braconidae) more strongly. However, in some cases, herbivore insects can minimize the volatiles when under pressure [37]. High aphid density can affect plant metabolism and may thereby change the volatile’s composition and affect the parasitoids. Florencio-Ortiz et al. [38] found that the total free amino acid was significantly reduced in pepper *Capsicum annuum* Linnaeus (Solanaceae) in response to the feeding of 200 individuals of the green peach aphid *Myzus persicae* Sulzer (Hemiptera: Aphididae). In addition, previous studies explained their behavioral results by assuming the reduction of volatiles resulted from increasing insect densities on plants [39,40,41]. However, these studies were not supported by chemical analysis. Furthermore, parasitoid females may also perceive other chemical cues that enable them to detect the presence of conspecific females, predators, and entomopathogens, thus avoiding competition or predation in the occupied patches [42,43,44,45,46].

The fact that aphids live in colonies may result in a positive encounter–dilution effect, increasing the patch residence time of female parasitoids [47]. This dilution effect is enhanced by the presence of exuviae (shed skins) on plants, which act as traps. Indeed, it has been proved that exuviae can provide protection to the aphid colony, as female parasitoids spend more time examining and stinging the exuviae, consequently reducing the efficiency of parasitoid attack on live aphid individuals [47,48]. Paradoxically, when aphid colony size increases, the dilution effect can be hampered by the increase in detectability, as the chemical cues emitted increase proportional to aphid number.

In this study, we used the tritrophic system: broad bean *Vicia faba* Linnaeus (Fabaceae)–pea aphid *Acyrthosiphon pisum* Harris (Homoptera: Aphidiidae)–parasitoid *Aphidius ervi* Haliday (Hymenoptera: Braconidae). *Aphidius ervi* has been studied mainly regarding behavioral bio-assays and attraction toward the aphid/plant complex [49,50,51,52]. We combined behavioral and chemical approaches to increase understanding of the bottom-up effects in plant–aphid–parasitoid interactions. According to the group vigilance hypothesis, we predicted that increasing the density of the aphid *A. pisum* would reduce the attraction of *A. ervi* parasitoid females. We investigated whether increasing the density of the aphid would manipulate the plant host to decrease the emitted volatiles and thus decrease the attractiveness for the parasitoid. We collected and identified the volatiles emitted from infested plants. Finally, it may be possible that the presence of a parasitoid female within the aphid colony on the plant may play a role in the attraction of its conspecifics. We therefore predicted that the presence of conspecific females would not advantage the attraction of other females.

## 2. Materials and Methods

### 2.1. Plant and Insect Rearing

The broad bean (*V. faba*) seeds were sown in trays filled with sphagnum peat moss growing medium and maintained in a growth chamber, insect-free, at 25 ± 1 °C, 75 ± 1% RH under a photoperiod 16L:8D. After 15 days, seedlings were transplanted individually into plastic pots and covered with a net mesh to avoid any infestation by insects.

The pea aphid, *A*. *pisum*, were collected in a pea field (Louvain-la-Neuve, Belgium) and then reared on *V. faba* in a mesh cage box (75 × 75 × 75 cm) and maintained in a growth chamber at 20 ± 1 °C and photoperiod 16L:8D, with fresh plants provided regularly.

About 500 mummies of the parasitoid *A. ervi* were obtained from Viridaxis SA (Belgium) and maintained in the laboratory on *A. pisum* colonies in a growth chamber at 20 ± 1 °C and photoperiod 16L:8D and humidity 70% ± 10. The experiments were commenced after two generations in the laboratory conditions.

For the experiments, *A. ervi* mummies were collected and isolated individually in Eppendorf^®^ tubes with a drop of honey. They were checked daily for the emergence of adults.

### 2.2. Bio-Assay Behavioral Experiment

A 14 cm long, glass Y-tube olfactometer was used, with an inner diameter of 1 cm and two 10 cm arms. Each front arm of the Y-tube olfactometer was connected to a glass cylinder containing a plant, either infested or non-infested. Both plants were placed in glass Erlenmeyer flasks sealed with Parafilm^®^ to avoid any odors from the growth media. The olfactometer was placed in a plexiglass box with the two external walls covered by paper sheets to avoid any external visual cues for the parasitoids. The plants were then placed in a Pyrex cylindrical chamber (d = 20 cm, H = 30 cm) connected with an airflow system. Upstream of the chamber, the airstream was adjusted to 1.2 L/min using an airflow meter, and the air was cleaned with activated charcoal.

All bio-assays were carried out between 10:00 a.m. and 6:00 p.m. in a room at 20.5 °C ± 1.5 °C, relative humidity 33% ± 2.3%, and under a 7500-lux light source. In order to avoid any asymmetrical bias, odor sources were alternated at each replication. The order in which treatments were tested was randomly modified each day to eliminate any time variation. After each replication, all glassware was scrupulously washed with an alkaline lab detergent (RBS 25), rinsed with deionized water, and oven-dried at 60 °C for 30 min.

Female parasitoids were introduced individually into the entrance of the Y-tube olfactometer and observed for a maximum of 15 min (900 s). Females were deemed to have made a final choice when they reached the end of either arm, at which point the test was stopped. Females that did not make any choice within 15 min were excluded. The total time taken to reach the end of the arm was recorded. Changing choices from one arm to another was considered a hesitation, and these rates were calculated as the number of hesitating females divided by the total number of females for each experiment. The experiment was recorded using the event recorder “The Observer” (Noldus Information Technology, Wageningen, The Netherlands).

### 2.3. Experiment 1: Effect of Aphid Density on Parasitoid Behavioral Choice

Five series of experiments were carried out to determine the influence of host density on the attraction of the female parasitoids toward aphid-infested plants compared to non-infested plants. Fifteen-day-old broad bean plants were infested with aphid individuals (larvae stage 2) 72 h before the experiments, with the following densities per plant: low (10 and 30 individuals), intermediate (50 individuals), and high (100 and 200 individuals). A total of 30 one-day-old females (1 female tested = 1 replicate) per treatment were tested. Five replicates were carried out per day for each treatment considered, for six days.

### 2.4. Experiment 2: Detectability of a Female Conspecific on a Host Colony

The consequence of a conspecific female parasitoid present on the aphid colony was tested using the same design as Experiment 1. Two series of experiments were set up: (1) a non-infested plant vs. a non-infested plant with one female parasitoid. A total of 30 one-day-old females were tested. (2) an infested plant with 30 aphids and one female parasitoid vs. a non-infested plant with a female parasitoid. A total of 20 one-day-old females were tested.

### 2.5. Chemical Extraction and Gas Chromatography–Mass Spectrometry (GC–MS) Analysis

The volatiles emitted from plants were collected from both aphid-infested and non-infested plants. Two densities of aphid were used (intermediate = 50 individuals, and high = 100 individuals). The volatiles were collected at the outlet of the glass cylinders by pumping the air through hand-made cartridges containing Super Q adsorbent (Alltech) [53] using vacuum pumps (Escort Elf Air Sampling Pump, Sigma–Aldrich). Two cartridges were set up in tandem to detect any loss of chemicals by breakthrough from the first cartridge. Airflow rates were adjusted at 400 mL/min through the traps. Four extractions (each extraction constituted three elutions) were carried out for non-infested plants and each density of aphid infested plants (intermediate and high), 12 extractions in total (3 × 4 replicates). The pumps circulated at a rate of 400 mL/min of air for 20 h. Each cartridge was then eluted three times with 250 µL of a solvent mixture (80:20, *n*-hexane (VWR):diethyl ether (Merck)). Next, 30 µL of n-butylbenzene (0.1 µg/µL) was added to each vial as an internal standard. Each elution was collected in a Teflon-capped vial, and samples were stored at 4 °C until analysis. Analyses were carried out within 2 days after the elution to avoid any chemical degradation.

Separation and identification of the organic compounds were conducted using an Agilent 6890N gas chromatograph coupled with an Agilent 5973N mass selective detector equipped with an HP-5 (Agilent) capillary column (30 m × 0.25 mm; I.D.: 0.25 µm film thickness). The oven temperature program was initiated at 40 °C, held for 5 min, then raised first at 10 °C/min to 230 °C, raised in a second ramp at 30 °C/min to 280 °C with a final hold at this temperature for 10 min. The carrier gas was He, maintained at a constant flow rate of 1.2 mL/min. Injection volume was 1 µL in splitless mode at an injection temperature of 280 °C. Interface temperature was maintained at 280 °C. Mass Spectrometry (MS) detection was performed with electron impact (EI) mode at 70 eV by operating in the full-scan acquisition mode in the 30–450 amu range. Identification of the volatile compounds was performed by comparing the mass spectra with those from the Wiley 275L spectral library. The compounds were identified by comparing their GC retention times with those of authentic standards.

### 2.6. Statistical Analysis

Data of behavioral and chemical results were not normally distributed. The preferences of parasitoid females regarding infestation type (aphid-infested vs. non-infested plants) and aphid densities were analyzed using Generalized linear model (GLM) with infestation type and individual density as fixed factors (quasibinomial response and logit link). To verify the probability of committing a Type-II error [54], we conducted a simulated power analysis for the GLM using the pwr package and pwr.f2.test function [55]. Significant results at *p* < 0.05 were followed by multiple comparison using the multcomp package and glht function [56]. The time taken for females to make a decision and reach the end of an arm was analyzed using GLM (Gamma distribution) with infestation type and aphid density as fixed factors. Time data were presented by the median and interquartile range (IQR). The hesitation rate of females between the choices was analyzed using GLM with choice option and aphid density as fixed factors (quasibinomial response and logit link) and data were presented as rate ± SE. For the two series of experiments regarding the presence of conspecific females, the preference of females in the Y-tube olfactometer and the time taken to reach the end of an arm were analyzed using the Mann–Whitney *U* test (MW *U*). Time data were presented as the median with (IQR). Since we had two separate series of experiments, the hesitation rates of females were presented as rate ± SE without a statistical test. The value of volatile blends was analyzed using the non-parametric ANOVA test (Kruskal Wallis KW) with individual density (non-infested, 50 and 100 aphid density) as a fixed factor, followed by the Mann–Whitney *U* test for pairwise comparisons, and data were presented as the median with (IQR). The analyses were conducted using the statistical software R version 4.0.5 (31 March) [57].

## 3. Results

### 3.1. Experiment 1: Effect of Host Density on A. ervi Response

**Figure 1:**
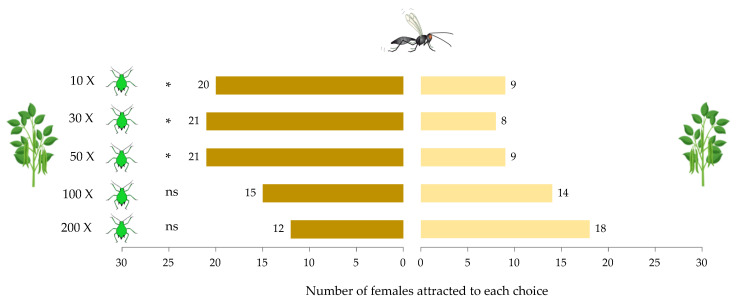
Response of *Aphidius ervi* females toward non-infested vs. aphid-infested plants with various aphid densities (10, 30, 50, 100, and 200 individuals of *Acyrthosiphon pisum*). (*) represents significant interaction between aphid density and type of infestation.

*Aphidius ervi* females significantly preferred aphid-infested plants compared to non-infested plants (F _1,54_ = 14.26, *p* < 0.001). Overall, the density of *A. pisum* individuals was found to have no impact on the attraction of parasitoid females (F _4,55_ = 0.04, *p* = 0.99). However, a significant interaction was found between infestation type and density of aphid individuals (F _4,50_ = 4.84, *p* = 0.002), where parasitoid females were significantly more attracted to the low and intermediate densities of aphid-infested plants (10, 30, and 50 individuals) compared to high aphid densities (100 and 200 individuals) (Figure 1). Based on the interaction between the two factors, an effect size estimate of *η*^2^ = 0.24 was calculated, and with α = 0.05, the power analysis achieved to 81%.

The time taken by females to make a final decision and reach the end of an arm did not vary significantly between aphid-infested plants and non-infested plants (F_1,139_ = 2. 71, *p* = 0.10) and did not vary among various densities of aphid individuals (F _4,140_ = 0.51, *p* = 0.72). No significant interaction was found between infestation type and individual density (F_4,135_ = 2.01, *p* = 0.09) (Table 1).

The hesitation rate of *A. ervi* females was generally low (Table 2). It did not vary significantly between the two choices (F_1,20_ = 1.95, *p* = 0.18) or among various aphid densities (F_4,16_ = 1.82, *p* = 0.19). No significant interaction was found between the two factors (F_4,12_ = 0.33, *p* = 0.84).

### 3.2. Experiment 2: Detectability of a Female Conspecific on a Host Colony

**Figure 2:**
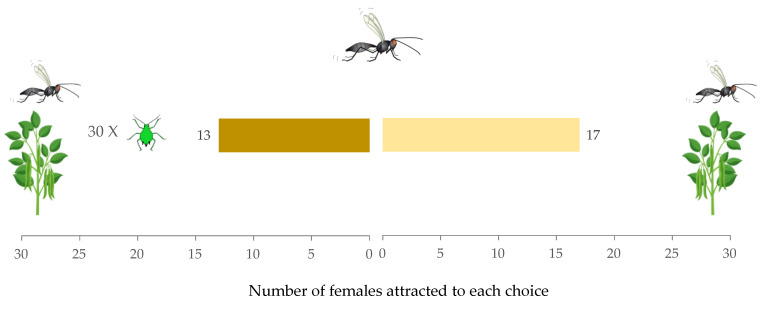
Response of *Aphidius ervi* females in a Y-tube olfactometer toward aphid-infested plant with conspecific female vs. non-infested plant with conspecific females.

When the effect of the presence of conspecific females with aphid-infested versus non-infested plants was analyzed, it was found that female parasitoids in the Y-tube olfactometer had no preference (W = 15, *p* = 0.68) (Figure 2). The time taken for females to make a decision between the two choices, aphid-infested plants = 17.97 s (IQR = 18.32) and non-infested plants = 28.23 s (IQR = 27.04), did not vary significantly (W = 217, *p* = 0.28). The hesitation rate of females was (0.20 ± 0.07).

**Figure 3:**
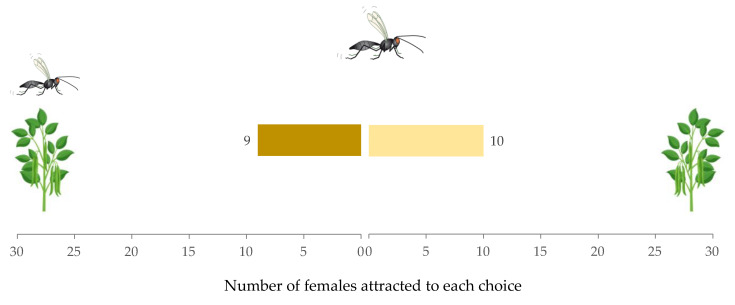
Response of *Aphidius* *ervi* females in a Y-tube olfactometer toward non-infested plant with conspecific female vs. non-infested plant.

Similarly, females given a choice between non-infested plants with a conspecific female present versus only non-infested plants showed no preference (W = 7, *p* = 0.88) (Figure 3). The time taken to make a decision between the two choices (29.75 s (IQR = 26.42), 19.92 s (IQR = 9.19), respectively) did not vary significantly (W = 46, *p* = 0.96). The hesitation rate of females was (0.25 ± 0.09).

### 3.3. Volatile Blends

Both non-infested plants and aphid-infested (intermediate = 50 individuals and high = 100 individuals) plants produced chemical volatiles. A total of six organic compounds were collected altogether (Table 3). Most emitted volatile compounds did not vary significantly in concentration among the plant treatments (Table 3), with two exceptions, 6-methyl-5-hepten-2-one and cis-jasmone. The former, 6-methyl-5-hepten-2-one, had significantly higher concentration in emissions from plants with intermediate aphid infestation density, compared to plants with high-density infestations and non-infested plants (Table 3). Emissions of cis-Jasmone were significantly higher from plants with intermediate density aphid infestations and non-infested plants than from plants with high-density infestations (Table 3). At intermediate infestation density, 26% (451 ng h^−1^) of the whole extracted blend was 6-metyl-5-hepten-2-one, followed by cis-jasmone (23.4%, 403 ng h^−1^) and E-2-hexanal (16.5%, 287 ng h^−1^). The total concentration of the volatile compounds varied significantly among the treatments, with higher total concentration emitted from plants with intermediate density infestation, followed by non-infested plants, and the lower total concentration emitted from plants with high density infestation (Table 3).

## 4. Discussion

The bottom-up effect is key to understanding how various species interact among multi-trophic levels in agroecosystems. By combining behavioral and chemical experiments, this study shows the bottom-up effects in a plant–aphid–parasitoid system. Consistent with many studies [58,59,60], our results overall showed that parasitoid females preferred infested plants over non-infested plants. However, this study underlines that low and intermediate aphid densities are more attractive to *A. ervi* females than higher densities of aphids. These results came from prudent optimization and were supported by the lower *p* value and high value of power analysis. A power analysis showed that this study had 81% power to detect the significant interaction between infestation type and density of aphid. The lower *p* value and high power outlined that the effect size in our study was enough to detect the significant differences. Indeed, high power analysis decreases the probability of making a Type II error [54].

The generalist *A. ervi* appears to imitate specialist aphid parasitoids, which often prefer aphids in sparse colonies [61]. The lower attractiveness of higher aphid densities to the parasitoids may result from factors such as aphid colony structure, increased host vigilance, and host defense mechanisms [62]. It could also be due to risk-spreading (the “bet-hedging strategy”) [63,64]. According to this strategy, parasitoids aim to attack a larger number of small colonies, rather than fewer big ones, laying eggs in various discrete patches to avoid the overcrowding of offspring and the high mortality of parasitized aphids at high density. Interestingly, Ives and Settle [65] found a high mortality rate for *A. ervi*-parasitized aphids before mummy formation at high population densities of *A. pisum*.

Consistent with our results, Cascone et al. [39] demonstrated that the parasitoid *Diaeretiella rapae* McIntosh (Hymenoptera: Braconidae) significantly preferred plants of radish and black mustard infested by *M. persicae* and *Brevicoryne brassicae* Linnaeus (Hemiptera: Aphididae), respectively, to non-infested plants, but only at low aphid infestation (25 vs. 100 individuals). Guerrieri et al. [51] showed that *A. ervi* was significantly attracted to *A. pisum*-infested broad bean with a minimum of 40 aphid individuals. On the other hand, previous studies on other parasitoids showed contradictory results. For instance, Yang et al. [66] showed that *Aphidius gifuensis* Ashmaed (Hymenoptera: Aphidiidae) was only attracted to infested tobacco and oilseed rape with high levels of density (200 and 400 individuals) of the aphid *M. persicae*. However, Tan and Liu [60] demonstrated that *A. gifuensis* females were attracted to *M. persicae*-infested tomatoes with as few as 20 aphid individuals. The difference between the two studies could be related to the host plants. Indeed, the host plant plays a crucial role in attracting specific parasitoids and affects parasitoid foraging behavior [67,68,69]. We infer from the patterns of attraction demonstrated in our Y-tube olfactometer experiments, that the potential mortality of aphids due to parasitism by *A. ervi* would be inversely density-dependent (i.e., the risk of parasitism decreases with increasing aphid density) [70,71].

Parasitoid species can locate their hosts in a very complex agro-environment using indirect volatiles emitted from insect-infested plants [35,72]. It has been suggested that the qualitative composition of the volatile blend produced by non-infested plants is very similar to the one produced by aphid-infested plants. However, aphid-infested plants show a quantitative increase in the volatile compounds [31,73]. Interestingly, in our study, the only compound found at a higher concentration was the 6-methyl-5-hepten-2-one, emitted in higher concentrations from plants infested with intermediate aphid density (50 individuals), compared to higher aphid density (100 individuals) and non-infested plants. This compound has been shown to attract the parasitoid *A. ervi* to *A. pisum*-infested plants [53] and prompt its flight response [31]. The concentration of 6-methyl-5-hepten-2-one seems to increase with the duration of aphid feeding [53], and it would be interesting to analyze further the dynamics of emission in relation to aphid infestation. The differences in emission of 6-methyl-5-hepten-2-one among infested and non-infested plants could explain why *A. ervi* females preferred low and intermediate aphid densities over high aphid densities on plants. This preference implies that parasitoid females have a capacity for discrimination of odor concentration. At the same time, it highlights the inverse density-dependent regulation of aphid-induced plant volatiles. Other volatile compounds, namely cis-jasmone and 3-hexenyl-acetate, were also emitted in higher concentrations. Birkett et al. [74] showed in a wind tunnel experiment that cis-jasmone is very attractive to the parasitoid *A. ervi* and the predator *Coccinella septempunctata* Linnaeus (Coleoptera: Coccinellidae) and also activates the plant defense. It appears that each parasitoid is attracted to one or more specific volatiles emitted from plants but not necessarily to the relative concentration of their mixture. For instance, Sun et al. [75] found that the parasitoid *Campoletis chlorideae* Uchida (Hymenoptera: Ichneumonidae) is attracted to two volatile compounds separately, *cis*-jasmone and *cis*-3-hexenyl acetate, but not when they are mixed. This study confirms that *A. ervi* parasitoids react to a specific volatile cue from plants at low and intermediate density of aphid infestation and supports the speculation that herbivore insects may be able to reduce the emitted volatiles from plants [37] and thus lower the risk of parasitism. Baluška and Ninkovic [76] suggested that infested plants stop producing these volatiles when herbivore densities become higher. For the moment, the mechanism responsible for lowering specific compounds at high aphid density is unclear. In this study, we did not test for the presence of endosymbiotic bacteria. Frago et al. (2017) showed that plants infested by the aphid *A. pisum* carrying the facultative symbiont *Hamiltonella defensa* reduced the emission of induced plant volatiles and thus were less attractive to the parasitoid *A. ervi* females [77]. It would be interesting to study the relationship among the endosymbionts, aphid density, and the emission of the induced plant volatiles.

The prior presence of a female parasitoid in the aphid colony had no significant impact on the choice made by a conspecific female, with females selecting both occupied and unoccupied patches equally in both sets of experiments. The presence of a female parasitoid in the aphid colony might have changed the aphid emission of alarm kairomones. However, those compounds do not appear to be attractive to most natural enemies [78]. We assume that *A. ervi* females did not actively avoid prior presence, perhaps because they did not perceive any difference. However, we did use only one female in the experiments, and maybe there would have been a various response to a greater number. It is also possible that conspecifics might be perceived as an indication of aphid presence [79], which the risk of super-parasitism would counter. Indeed, the presence of conspecifics in the same patch could increase super-parasitism [80]. The avoidance or non-avoidance of conspecific females over long distances has been studied in many parasitoid species. It has been shown that females of the parasitoid *Venturia canescens* Gravenhorst (Hymenoptera: Ichneumonidae) avoid their conspecifics only when the density reaches 20 females on the patch [43]. Janssen et al. [45] demonstrated that the *Drosophila* parasitoid *Leptopilina heterotoma* Thomson (Hymenoptera: Eucoilidae) avoided the host patch in the presence of five individuals, both with conspecifics and its heterospecific, *L. clavipes* Hartig. Avoidance of conspecifics by parasitoid females depends mainly on the density of its conspecifics indicating competition. However, the non-avoidance of conspecific competitors has been also reported [81]. Some parasitoids, including *A. ervi*, can discriminate between self-parasitized and conspecific-parasitized hosts [82], as well as between parasitized and un-parasitized hosts [83]. For instance, Le Lann et al. [84] demonstrated that, when *A. ervi* females were given a choice, they avoided super-parasitism and preferred to lay eggs on un-parasitized rather than parasitized hosts.

To conclude, combining behavioral and chemical approaches enabled a more holistic deciphering of plant–aphid–parasitoid systems. Low and intermediate aphid densities appeared to be more attractive to parasitoids, corresponding to a higher concentration of 6-methyl-5-hepten-2-one. At high densities of aphid infestation, the concentration of this compound drops, indicating that aphids in large colonies may be capable of manipulating plants to reduce the rate of detection by parasitoid females, reducing the risk of parasitism. However, further investigations would be essential to identify the feeding behavior of aphids at various densities on plants, perhaps combining volatile emission studies with electrical penetration graphs. In practice, if parasitoids are not attracted to high-density aphid infestations, it could negatively impact the potential for biological control. Therefore, we recommended the release of parasitoids early in the season when aphid density is still low in the field; otherwise, high aphid density may reduce the activity of the parasitoids.

## Figures and Tables

**Table 1:** The time taken (Median with IQR) in seconds by *Aphidius ervi* females to make a decision between aphid-infested and non-infested plants. No significant differences were found.

Time Taken in Seconds by Parasitoid Females to Make a Decision		
Individual Density	Aphid-Infested Plants	Non-Infested Plants
10	36.22 s (49.16)	30.85 s (48.82)
30	37.69 s (18.23)	16.34 s (42.20)
50	40.26 s (43.53)	17.14 s (16.83)
100	23.03 s (16.61)	45.28 s (78.61)
200	29.56 s (42.75)	38.30 s (29.65)

**Table 2:** The rate of choice hesitation of *Aphidius ervi* females between aphid-infested and non-infested plants. Data are presented as rate ± SE. No significant differences were found.

The Rate of Choice Hesitation of Parasitoid Females between the Two Choices		
Individual Density	From Aphid-Infested to Non-Infested Plants	From Non-Infested to Aphid-Infested Plants
10	0.10 ± 0.05	0.20 ± 0.07
30	0.07 ± 0.05	0.20 ± 0.07
50	0.07 ± 0.05	0.10 ± 0.05
100	0.03 ± 0.03	0.03 ± 0.03
200	0.23 ± 0.07	0.03 ± 0.03

**Table 3:** Volatile organic compounds from aphid-infested plants (50 and 100 individuals) and non-infested plants. Data are presented as median (IQR). Small letters represent significant difference among treatments.

VOCs ng/μL	Non-Infested	Aphid Infested		Statistical Analysis
	0 Individuals	50 Individuals	100 Individuals	
6-methyl-5-hepten-2-one	1.03 (0.95) b	3.81 (0.64) a	0.35 (0.25) b	KW test: χ^2^ = 9.26, df = 2, *p* = **0.009**
cis-jasmone	1.84 (0.68) a	3.25 (1.25) a	0.32 (0.04) b	KW test: χ^2^ = 8.89, df = 2, *p* = **0.01**
cis-3-hexenol	0.77 (1.15) a	2.61 (2.46) a	0.11 (only one record)	MW U test: W = 6.5, *p* = 0.77
cis-3-hexenyl-acetate	1.88 (0.47) a	3.17 (1.42) a	0	MW U test: W = 4, *p* = 0.67
linalool	0.46 (0.03) a	1.37 (0.37) a	0	MW U test: W = 4, *p* = 0.33
E-2-hexenal	0.87	1.43	0	No test
Total	5.22 (1.40) b	12.49 (2.92) a	0.53 (0.62) c	KW test: χ^2^ = 9.84, df = 2, *p* = **0.007**

## Data Availability

The data presented in this study are available upon request from the corresponding author.
